# A Condition Evaluation Simplified Method for Traction Converter Power Module Based on Operating Interval Segmentation

**DOI:** 10.3390/s23052537

**Published:** 2023-02-24

**Authors:** Lei Wang, Mingchao Zhou, Zhonghao Dongye, Yanbei Sha, Jingcao Chen

**Affiliations:** 1 School of Electrical Engineering, Beijing Jiaotong University, Beijing 100044, China; 2 China Railway Shanghai Group Co., Ltd., Shanghai 200071, China

**Keywords:** operating interval segmentation, condition evaluation simplified method, power module of traction converter, temperature and fatigue stress, lifetime assessment

## Abstract

In the actual operation of urban rail vehicles, it is essential to evaluate the condition of the traction converter IGBT modules. Considering the fixed line and the similarity of operation conditions between adjacent stations, this paper proposes an efficient and accurate simplified simulation method to evaluate IGBT conditions based on operating interval segmentation (OIS). Firstly, this paper proposes the framework for a condition evaluation method by segmenting operating intervals based on the similarity of average power loss between neighboring stations. The framework makes it possible to reduce the number of simulations to shorten the simulation time while ensuring the state trend estimation accuracy. Secondly, this paper proposes a basic interval segmentation model that uses the operating conditions as inputs to implement the segmentation of the line and is able to simplify the operation conditions of entire line. Finally, the simulation and analysis of the temperature and stress fields of IGBT modules based on segmented intervals completes the IGBT module condition evaluation and realizes the combination of lifetime calculation with actual operating conditions and internal stresses. The validity of the method is verified by comparing the interval segmentation simulation with actual test results. The results show that the method can effectively characterize the temperature and stress trends of traction converter IGBT modules in the whole line, which could support the fatigue mechanism and lifetime assessment reliability study of IGBT modules.

## 1. Introduction

Since the traction converter system provides power for urban rail vehicles, improving its reliability has a positive significance for safety and maintenance [1,2]. IGBT power modules have been widely used in traction converters due to their good physical properties [3,4,5]. However, owing to the influence of various working conditions and complex environments during the operation of urban rail vehicles, IGBT modules are subjected to electrical, thermal, and vibration fatigue stresses, which accelerate the fatigue failure process of IGBT modules and cause them to become the main limitation affecting the operational reliability of urban rail vehicles [6,7]. Therefore, it is important to evaluate the condition of power modules for extending the effective running time, reducing the failure rate of train operation, and ensuring safe operation [8,9].

When an urban rail vehicle operates under a single working condition, the junction temperature of IGBT module rises and falls under the repeated action of the continuous Pulse-Width-Modulation (PWM) driver signals. When the heat generation by IGBT and the dissipation by heat-sink form a balance, the junction temperature of the IGBT module fluctuates periodically around a stabilized equilibrium point. Hence the junction temperature value at the equilibrium point is accordingly higher. However, owing to the change in the working conditions (i.e., periodical acceleration and deceleration in accordance with the route map) of urban rail vehicles during operation, the original heat balance can be broken, and then a new heat balance point is generated [10]. Therefore, the average junction temperature of the IGBT module would repeatedly change during operation. During the repeated heating and cooling process, the internal materials of the IGBT module are repeatedly impacted by thermal stress, which leads to a final fatigue failure. Simultaneously, vibration will fatigue the packaging structure of electronic devices [11,12]. IGBT modules of the inverter unit in an urban rail vehicle are installed perpendicular to the ground, and the vertical vibration will make the IGBT bonding wires and solder layer subject to shear stress when the vehicle crosses turnout, resulting in slight relative displacement and deformation between the layers inside the module. Under the repeated impact of the vibration stress, the IGBT module may have cracks and fractures at the connection interface, which accelerates the fatigue failure of the module [13,14]. Urban rail vehicles go through many stations from the origin to the destination. The speed and load conditions of the vehicle between each station are not exactly the same, so the temperature and stress of the IGBT modules vary between stations. Considering that the stress and temperature of the IGBT module cannot be directly observed during the actual operation of the vehicle, in order to more comprehensively study the temperature and stress of the IGBT module during the operation in the whole line, it is necessary to simulate the vehicle operation conditions between each station in the whole line one by one.

The research on evaluating IGBT module conditions based on the task profile simulation currently includes electrothermal simulation and multi-physics field simulation based on the finite element model. The electrothermal simulation is based on the circuit model of the converter and the thermal network model. The junction temperature of the task profile is obtained by changing the load power and calculating the power loss of IGBT devices under different operation conditions. The obtained junction temperature information is combined with the life model to be applied in the lifetime evaluation of IGBT devices. Ref. [15] combines the high-speed train operation diagram to reflect the train operating condition changes to the rectifier DC side current changes, and then clarifies the changes of the IGBT electrical characteristics. Finally, it obtains the junction temperature changes of the IGBT based on the line task profile and analyzes the IGBT lifetime by the rain flow counting method (RFCM) and the life model. Ref. [16] comparatively studied power loss estimation models with two different time resolutions based on two electric vehicle (EV) standard driving cycle profiles. It used junction temperature information to predict the lifetime of power devices by the RFCM and the life model, and finds that the differences in the EVs and the selection of the power loss model affect the lifetime prediction results. Ref. [17] establishes an electrothermal simulation model of the urban rail vehicle traction drive system, obtains the power loss and junction temperature variation curves of the IGBT based on the task profile of the urban rail vehicle line, calculates the lifetime of the IGBT by the RFCM and the cumulative damage model, and, finally, studies the traction converter control strategy, which can improve the reliability of traction converters. This method can clearly describe the fluctuation of the IGBT junction temperature calculated by the thermal network model under actual operation conditions, and the simulation speed is relatively fast. However, the thermal network model established by this method does not effectively reflect the temperature changes at different locations inside the IGBT, and the corresponding stress cannot be clarified, which is not conducive to the study of the fatigue failure mechanism of the IGBT under actual operation conditions, and the lifetime obtained through the junction temperature information cannot reveal the influence of internal stress action on the lifetime. In addition, in the process of analyzing the lifetime of the IGBT in rail transportation areas, the low cycle junction temperature fluctuations caused by the acceleration, idling, and deceleration speed conditions between each station lead to a relatively large calculation of the number of cycles analyzed by the RFCM. In order to study the internal stress action mechanism of IGBT modules in converter systems, the use of finite element simulation is an accepted method [18,19]. Ref. [20] analyzes the temperature and stresses between different materials and layers using an IGBT finite element model (FEM) after correcting for loss errors, and also investigated the effect of different defects in the solder layer on the temperature and stresses of the IGBT. Ref. [21] investigates the effects of the clamping area on collector deformation, temperature, and stress distributions using FEM for Press-pack IGBTs (PP-IGBTs), and also analyzed the effect of heat-sink thickness to maximize the stress evenness of the terminal PP-IGBT and reduce the overall length of the stack system. It can be concluded that the finite element simulation can accurately reflect the temperature and stress of IGBT devices, and can be used to study the effect mechanism of different parameters on temperature and stress. However, finite element simulation requires more resources and a longer simulation time, both of which limit the application of simulation for scenarios with a large number of working conditions. At the same time, the actual operation of urban rail vehicles generates vibration, and the action mechanism of vibration stress on IGBT modules in urban rail vehicles is less analyzed in existing studies, and the combination of finite element simulation and actual operation conditions requires further research.

Therefore, the current problems can be summarized as follows: (1) The internal temperature and stress of power module can be obtained from the FEM, however, the long simulation time leads to low efficiency [22]; (2) Existing lifetime models have rarely been validated for accuracy under actual operation conditions [23], and internal stress effects are often neglected in the condition and lifetime estimation process. Limited by the above-mentioned problems and the number of urban rail vehicle stations [24], it is difficult to specify the conditions of the power module in the whole line. Therefore, this paper proposes a simplified condition evaluation method based on operating interval segmentation. The contributions of the method can be summarized as follows: (1) This paper proposes a simplified framework for the process of condition evaluation, which can reduce the number of simulations and the amount of computation while ensuring accuracy. (2) This paper proposes a basic model of line interval segmentation based on operation conditions which can simplify the operation conditions. (3) This paper clarifies the temperature and stress variation trend of the power module of the whole line through the simulation of each interval, and calculates the lifetime.

The structure of this paper is organized as follows. Section 2 introduces the process of establishing the digital simulation model, including the electrical simulation model of the traction converter system, the power loss calculation model, the vibration analysis of the urban rail vehicle, and the FEM. Section 3 proposes a simplified digital simulation method to segment the line interval based on the established power loss data space and the actual operation conditions of the whole line of the urban rail vehicle; introduces the digital simulation analysis process based on the proposed method; and carries out power loss simulation, temperature, thermal stress, and vibration stress simulations and analyses. Section 4 introduces the validation process of this method based on the test platform built, and this section also introduces the process of applying the stress simulation results based on this method to the IGBT lifetime calculation. Section 5 concludes this paper.

## 2. Digital Simulation Model of Power Module

The fatigue stresses of IGBT modules in urban rail vehicles mainly include thermal stresses and vibration stresses. In order to establish the fatigue stress digital simulation model of IGBT module, its electric–thermal characteristics and vibration characteristics need to be modeled and analyzed.

### 2.1. Power Loss Model of Power Module

**Figure 1:**
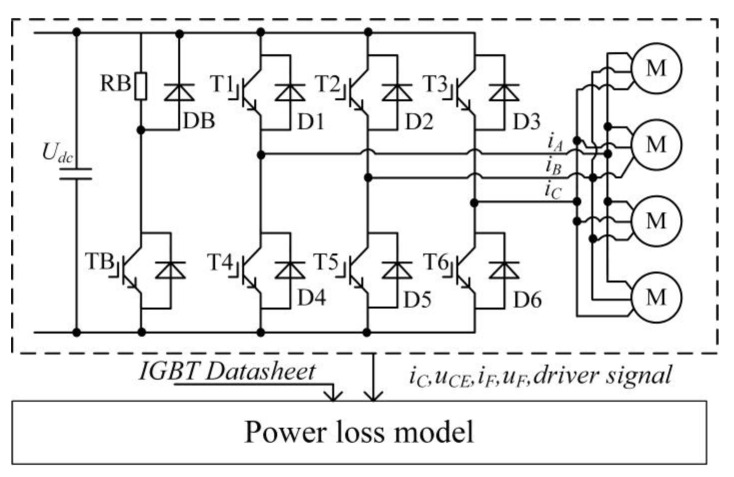
Power loss simulation topology of traction converter for urban rail vehicles.

The power loss simulation topology of the traction converter for urban rail vehicles is shown in Figure 1. The IGBT and freewheeling diode (FWD) generate power losses during the actual operation. The loss of IGBT mainly includes the on-state loss, turn-on loss, and turn-off loss, and the loss of the FWD mainly includes the on-state loss and reverse recovery loss.

Considering that the three phases of the traction converter are in equilibrium and each phase of the power module generates the same power loss during the positive and negative half fundamental-wave cycle, the power loss generated by the traction converter is calculated by the power loss generated by one phase. In this paper, the power loss model of the traction converter is established by the power loss of the IGBT and the FWD in phase A during the positive half fundamental-wave cycle based on the SVPWM modulation method.

Setting the power factor angle α to [0,π/6), the sector *N* through which the voltage space synthesis vector passes is V-VI-I-II, and the duty cycle corresponding to each sector is $\delta_{N}$. Therefore, the on-state power loss $P_{cond(T1)}$ of the IGBT T1 is expressed by Equation (1), and the switching power loss $P_{sw(T1)}$ is expressed by Equation (2), when the current is positive (half the fundamental-wave cycle).

(1) $$\{\begin{array}{l} u_{CE}(t)=V_{CEO}+i_{C}(t)\cdot R_{T}\\ V_{CEO}=V_{CEO25℃}+K_{VT}(T_{jT}-25℃)\\ R_{CE}=R_{CE25℃}+K_{rT}(T_{jT}-25℃)\\ \begin{array}{c} P_{\mathrm{cond}(T1)}=\frac{1}{2\pi }\int_{-\frac{\pi }{2}+\alpha }^{-\frac{\pi }{3}}u_{CE}(t)\cdot i_{C}(t)\cdot \delta_{5(T1)}d\omega t+\\ \;\;\frac{1}{2\pi }\int_{-\frac{\pi }{3}}^{0}u_{CE}(t)\cdot i_{C}(t)\cdot \delta_{6(T1)}d\omega t+\\ \;\;\frac{1}{2\pi }\int_{0}^{\frac{\pi }{3}}u_{CE}(t)\cdot i_{C}(t)\cdot \delta_{1(T1)}d\omega t+\\ \;\;\frac{1}{2\pi }\int_{\frac{\pi }{3}}^{\frac{\pi }{2}+\alpha }u_{CE}(t)\cdot i_{C}(t)\cdot \delta_{2(T1)}d\omega t \end{array} \end{array}$$

In Equation (1), $u_{CE}$ is the on-state voltage of IGBT T1, $i_{C}$ is the collector current of IGBT T1, $V_{CEO}$ is the threshold voltage of IGBT T1, $R_{CE}$ is the on-state resistance of IGBT T1, $K_{VT}$ is the on-state voltage temperature coefficient of IGBT T1, $K_{rT}$ is the on-state resistance temperature coefficient of IGBT T1, $T_{jT}$ is the junction temperature of IGBT T1, ω is the rotation angle frequency of the voltage space synthesis vector, and $\delta_{N}$ is the duty cycle of the sector where the voltage space synthesis vector is located.

Setting the power factor angle α to [0,π/6), the sector *N* through which the voltage space synthesis vector passes is V-VI-I-II, and the duty cycle corresponding to each sector is $\delta_{N}$. Therefore, the on-state power loss $P_{cond(T1)}$ of IGBT T1 is expressed by Equation (1), and the switching power loss $P_{sw(T1)}$ is expressed by Equation (2), when the current is positive (half the fundamental-wave cycle).

(2) $$\{\begin{array}{c} \begin{array}{c} E_{\mathrm{on}}=(5.87e^{-4}\times i_{C}(t)+795.84)\times \\ \;\;[(8.14e^{-5}\times T_{jT}-1.02e^{-2})\times i_{C}(t)+\\ \;\;1.04T_{jT}-128.9] \end{array}\\ \begin{array}{c} E_{\mathrm{off}\;}=(1.51e^{-4}\times i_{C}(t)+42.92)\times \\ \;\;[1.11+(2.22e^{-5}\times i_{C}(t)+1.13)(T_{jT}-125)] \end{array}\\ P_{\mathrm{sw}(T1)}=f_{\mathrm{sw}}\times \frac{1}{\pi }\times (E_{\mathrm{on}}+E_{\mathrm{off}}) \end{array}$$

In Equation (2), $E_{on}$ is the turn-on energy loss per pulse of IGBT T1, $E_{off}$ is the turn-off loss energy per pulse of IGBT T1, and $f_{sw}$ is the switching frequency of IGBT T1.

The on-state power loss $P_{cond(D4)}$ of FWD D4 is expressed by Equation (3), and the switching power loss $P_{sw(D4)}$ is expressed by Equation (4).

(3) $$\{\begin{array}{l} u_{F}(t)=V_{FO}+i_{F}(t)\cdot R_{F}\\ V_{FO}=V_{FO25℃}+K_{VD}(T_{jD}-25℃)\\ R_{F}=R_{F25℃}+K_{rD}(T_{jD}-25℃)\\ \begin{array}{c} P_{\mathrm{cond}(D4)}=\frac{1}{2\pi }\int_{-\frac{\pi }{2}+\alpha }^{-\frac{\pi }{3}}u_{F}(t)\cdot i_{F}(t)\cdot \delta_{5(D4)}d\omega t+\\ \;\;\frac{1}{2\pi }\int_{-\frac{\pi }{3}}^{0}u_{F}(t)\cdot i_{F}(t)\cdot \delta_{6(D4)}d\omega t+\\ \;\;\frac{1}{2\pi }\int_{0}^{\frac{\pi }{3}}u_{F}(t)\cdot i_{F}(t)\cdot \delta_{1(D4)}d\omega t+\\ \;\;\frac{1}{2\pi }\int_{\frac{\pi }{3}}^{\frac{\pi }{2}+\alpha }u_{F}(t)\cdot i_{F}(t)\cdot \delta_{2(D4)}d\omega t \end{array} \end{array}$$

In Equation (3), $u_{F}$ is the on-state voltage of FWD D4, $i_{F}$ is the current of FWD D4, $V_{FO}$ is the threshold voltage of FWD D4, $R_{F}$ is the on-state resistance of FWD D4, $K_{VD}$ is the on-state voltage temperature coefficient of FWD D4, $K_{rD}$ is the on-state resistance temperature coefficient of FWD D4, $T_{jD}$ is the junction temperature of FWD D4.

(4) $$\{\begin{array}{l} \begin{array}{c} E_{\mathrm{rr}}=(-0.0002011i_{F}^{2}+1.118i_{F}+416.6)\cdot \\ \;\;\;[(1.458i_{F}^{0.02591})\cdot (T_{jD}-125)] \end{array}\\ P_{\mathrm{sw}(D4)}=f_{\mathrm{sw}}\cdot \frac{1}{\pi }\cdot E_{\mathrm{rr}} \end{array}$$

In Equation (4), $E_{rr}$ is the reverse recovery energy per pulse.

### 2.2. Random Vibration Analysis

**Figure 2:**
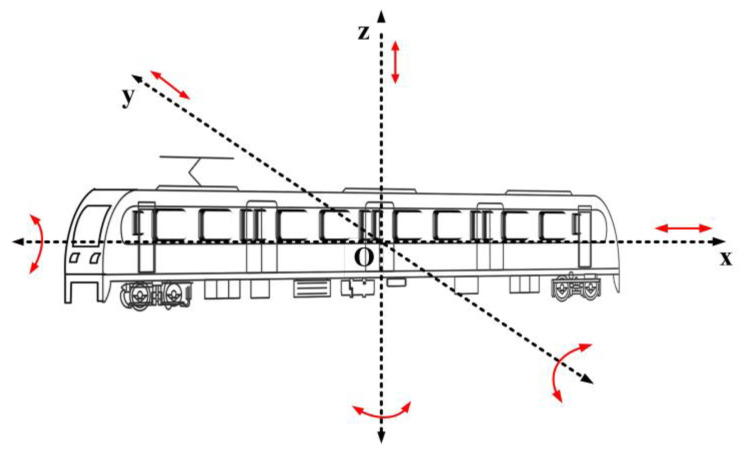
Schematic diagram of train vibration form.

Random vibration occurs during the operation of urban rail vehicles due to wheel–rail interaction caused by uneven track. The vibration of the vehicle body may relatively displace the layers of the IGBT module in the traction converter. The severe vibration will even cause the module to bend and the distribution parameters to change, which eventually causes the fatigue failure of the IGBT module. The form of the train vibration is shown in Figure 2. The vehicle body is placed in a Cartesian coordinate system, where the vehicle body is parallel to the *x*-axis and the center of gravity of the vehicle body is located at the coordinate origin O.

According to the vibration path, when the vehicle body vibrates, the vibration falls into three categories: longitudinal, transverse, and vertical vibration. Among such vibration forms, the acceleration of vehicle body vertical vibration is larger than its longitudinal and transverse acceleration [25,26], which makes the bonding wire and solder layer relatively susceptible to vibrations generated by vertical acceleration. Therefore, in order to improve the efficiency of the simulation, this paper focuses on the analysis of vertical random vibrations to elucidate the vibration stresses inside the IGBT module.

The vibration source is a random signal. As a complex random signal, the vibration wave of urban rail vehicles cannot be expressed by a specific functional expression in the time domain, and it needs to be converted from the time domain signal to the frequency domain signal by Parseval’s theorem, as shown in Equation (5).

(5) $$\int_{-\infty }^{+\infty }|x(t){|}^{2}dt=\frac{1}{2\pi }\cdot \int_{-\infty }^{+\infty }|F(\omega ){|}^{2}d\omega =\int_{-\infty }^{+\infty }S_{x}(f)df$$

In Equation (5), x(t) is the random signal, and F(ω) is the frequency domain signal; $S_{x}(f)$ is the power spectral density function, which represents the average power distribution of the signal in the frequency domain.

The average power of the signal is always conserved in the time and frequency domains by Parseval’s theorem: the area enclosed by $S_{x}(f)$ and f is equal to the average power of x(t). Depending on the physical quantity of x(t), which can be divided into displacement, velocity, and acceleration, the corresponding physical units of the $S_{x}(f)$ power spectral density also change. When the unit of x(t) is g, then the corresponding unit of $S_{x}(f)$ power spectral density is $g^{2}/\mathrm{Hz}$.

Since the train vibration waveform is not periodic and is usually only obtained over time, the frequency domain analysis method, which portrays the vibration characteristics more deeply, is more applicable. The power spectral density set in this paper is shown in Table 1 [27], and it is loaded into the vertical direction of the module for simulation tests.

### 2.3. Finite Element Simulation Model

In order to clarify the fatigue stress inside IGBT module, the FEM of the IGBT module (FZ1500R33HE3) and its heat-sink are established in this paper. The results of the dimensional measurements for the IGBT module entity are shown in Table 2.

The traction converter heat-sink in urban rail vehicles generally adopts air-cooled heat dissipation, ventilating between the fins and controlling the airspeed to achieve a good heat dissipation effect. The heat-sink dimensions used in this paper are shown in Table 3.

The interior of FZ1500R33HE3 entity consists of six liner cells (DBC solder layer and above) with eight chips in each liner cell (four IGBT chips and four FWD chips). In order to better ensure the accuracy of the finite element model calculation, the density, thermal conductivity, coefficient of thermal expansion, specific heat capacity, elastic modulus, and Poisson’s ratio of the material in each layer of the model are assigned according to the data in Table 4, and the default values of each parameter of metallic aluminum are chosen for the heat-sink parameters.

**Figure 3:**
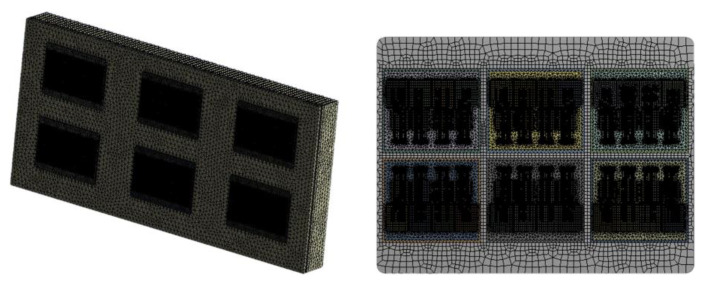
Mesh division of the model.

Considering that the traction converter contains six IGBT modules and a heat-sink, the six IGBT modules are placed evenly on the heat-sink in this paper, and the specific mesh division of the model is shown in Figure 3.

## 3. Condition Evaluation Simplified Simulation Method Based on OIS

### 3.1. Method Introduction

#### 3.1.1. Flow Chart of Simplified Condition Evaluation Based on OIS

**Figure 4:**
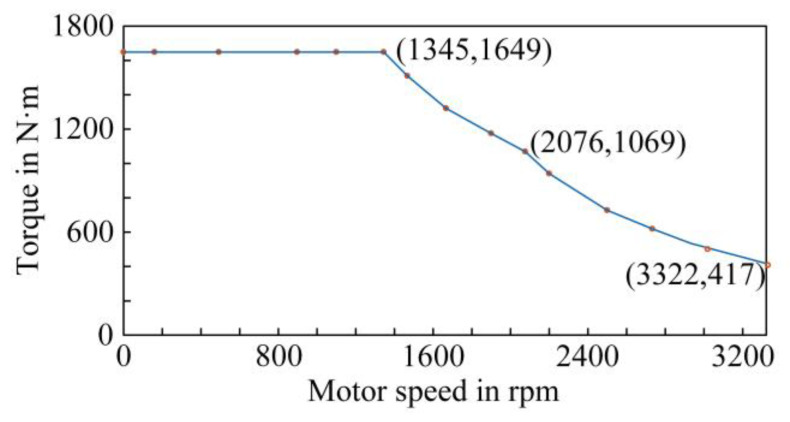
Traction characteristic curve of the urban rail vehicle.

Combined with the electric–thermal simulation model of the traction converter established in this paper and the traction characteristic curve of urban rail vehicles shown in Figure 4, the power loss of the IGBT and the FWD under different torque and speed conditions could be simulated, and the power loss data under different working conditions can be obtained.

**Figure 5:**
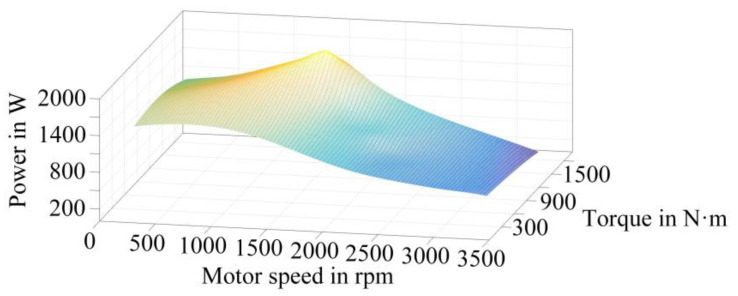
Power loss-speed-torque data space of IGBT.

**Figure 6:**
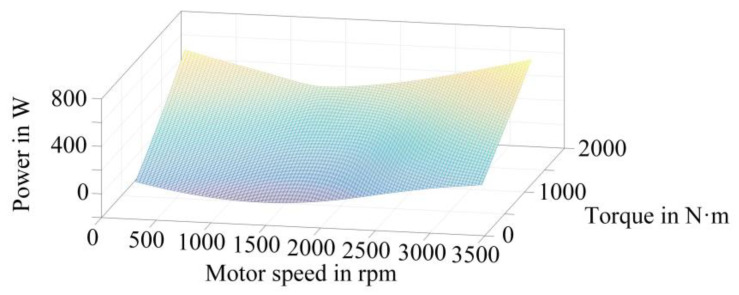
Power loss-speed-torque data space of FWD.

The obtained power loss data is processed to establish the power loss data space of the IGBT and the FWD in the traction converter, as shown in Figure 5 and Figure 6, respectively. The power losses corresponding to different operating conditions can be obtained from this data space.

**Figure 7:**
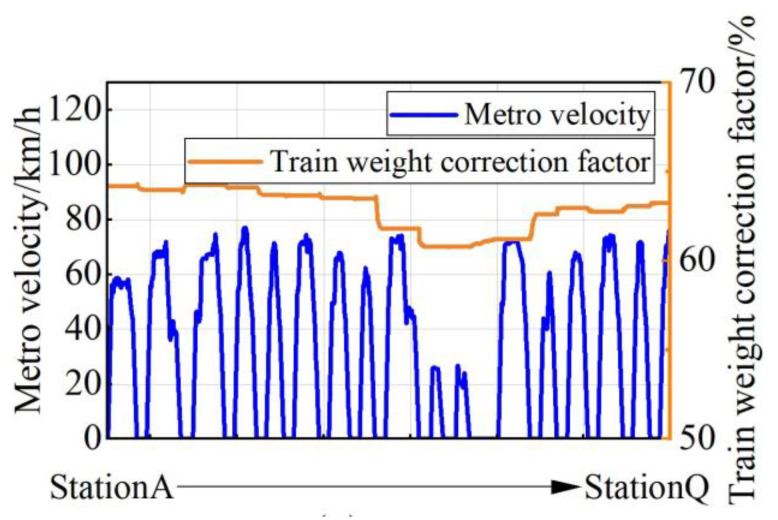
Train operation curves.

In this paper, the variation curves of the train speed and the train weight correction factor between each station are obtained by simulating the actual train operation curve, as shown in Figure 7. It can be seen that a total of 17 stations are included, and the speed is limited to below 80 km/h. The change in the vehicle weight correction factor reflects the increase or decrease in vehicle personnel. During the operation of the vehicle between the 17 stations, the speed and load conditions between two stations have differences, such as the lowest speed and the load during the arrival of the train at the end of the line; the speed is relatively high during the operation, and the load will change with the change in personnel.

For the fluctuation of the IGBT junction temperature, the low frequency component is mainly related to the operating conditions, the medium frequency component is mainly related to the fluctuation of inverter input and output power, and the high frequency component is mainly related to the inverter output frequency [28]. In order to clarify the stress distribution of IGBT modules in the whole line train and to evaluate the IGBT module lifetime, the traditional method needs to clarify the low-frequency variation of the IGBT module temperature and stress due to acceleration, deceleration, uniform speed, and load conditions between each station to achieve the IGBT module lifetime prediction, which is more accurate but less efficient.

Considering the different maximum speed and load of the vehicle operation between two stations, the average power loss of the IGBT module is also different. In order to accurately describe the condition and lifetime of IGBT modules in the whole line and to reduce the number of simulations, this paper segments the whole line into multiple operation condition intervals based on the similarity of the average power between adjacent stations. The simulation of temperature and stress fields is carried out with the average power of each interval as input, and the simulation results of each interval describe the overall trend of temperature and stress changes of the IGBT of the whole line. The results of the average power simulation are used to map the temperature fluctuation, which is a lower frequency component that describes the trend of the IGBT junction temperature of the whole line and could improve the simulation efficiency.

**Figure 8:**
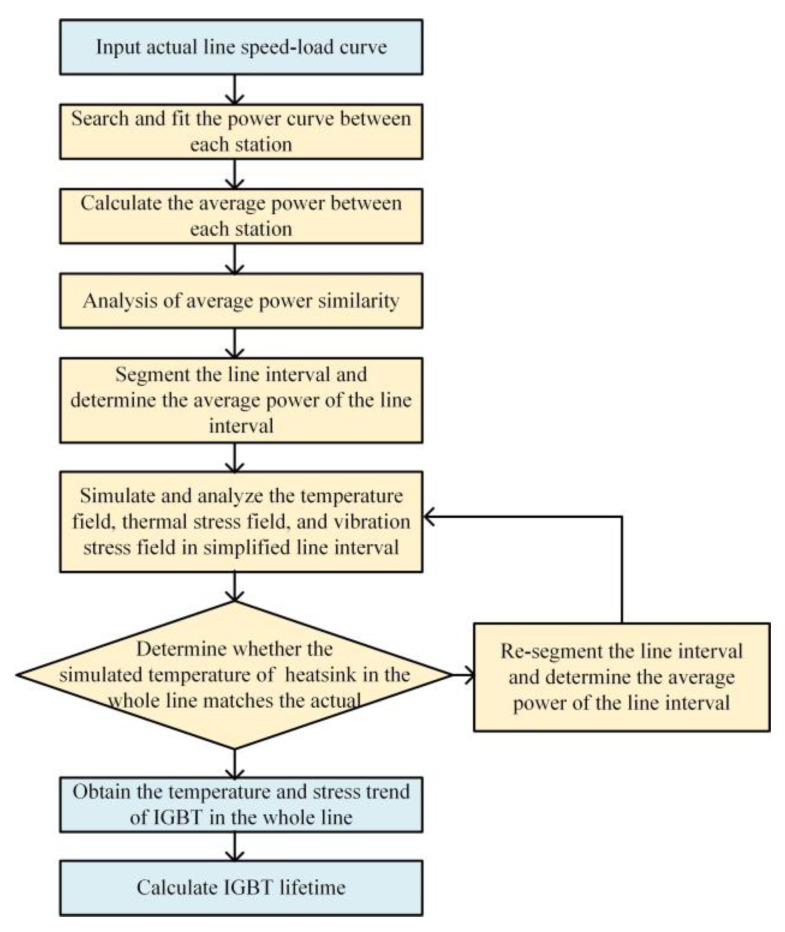
The flow chart of proposed method.

**Figure 9:**
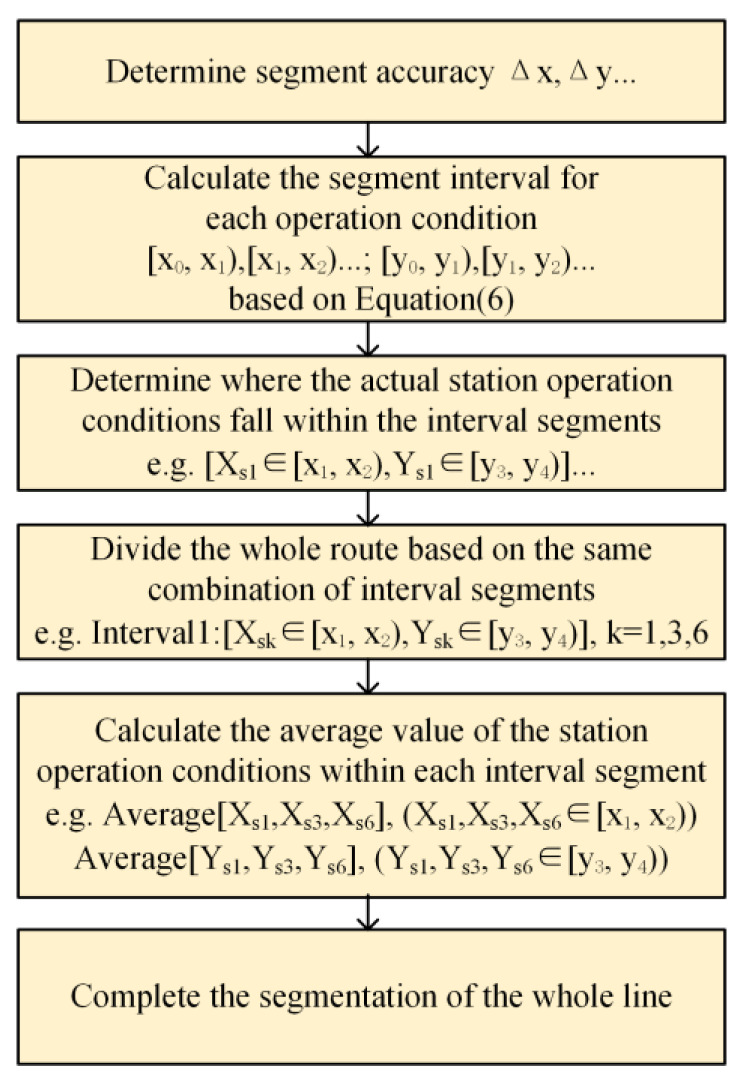
The flow chart of OIS of the whole line.

The flow chart of the proposed method is shown in Figure 8. To be able to meet the practical application, the proposed method takes the speed and load of the line as the input of the method, and the temperature trend and lifetime of the IGBT of the whole line as the output. First, a similarity analysis is performed and the segmentation of the whole line is completed based on the line speed and load conditions according to the process shown in Figure 9. Further, temperature and stress simulations are performed for each interval separately, and on the basis of verifying the accuracy of the model, the temperature and stress (thermal stress and vibration stress) results are used to clarify the temperature and stress variation pattern of the IGBT module of the whole line. Finally, lifetime of the IGBT module is calculated.

#### 3.1.2. OIS Mathematical Model with a Flow Chart

The flow chart of the whole line OIS is shown in Figure 9. In the process of segmenting the whole line, it is first necessary to determine the segmentation accuracy (segmentation criteria). The second step is to calculate all interval ranges corresponding to the segmentation accuracy and the actual operation conditions. The mathematical model for determining the range of all segment intervals corresponding to the operating conditions is given by Equation (6). The third step is to determine the interval range corresponding to the operation condition values [*X*_sk_,*Y*_sk_] for each station, forming the interval range combination $C_{sk}=[X_{sk}\in [x_{n},y_{m}),Y_{sk}\in [x\prime_{n},y\prime_{m})]$ for each station, where *k* is the station sequence number. In the fourth step, the stations of the same interval range combination *C_s_* are used as a segment interval (Interval l) in the whole line, where l is the segment interval sequence number of the line Interval l. The fifth step is to calculate the average value of each type of operating condition for each segment interval. Finally, once the number of segment intervals and the corresponding operation condition values have been determined, the whole line segmentation can be achieved.

(6) $$\left\{ \begin{array}{l} x_{n}=x_{0}+n\Delta x\;(\Delta x>0,n=1,2\ldots N)\\ x_{N-1}\leq \max[X_{s}]\leq x_{N}\;\\ y_{m}=y_{0}+m\Delta y\;(\Delta y>0,m=1,2\ldots M)\\ y_{M-1}\leq \max[Y_{s}]\leq y_{M}\;\\ \ldots \end{array}\right.$$

In Equation (6), $x_{n}$ represents the calculated value of operation condition 1 and $y_{n}$ represents the calculated value of operation condition 2. $x_{0}$, $y_{0}$ are the minimum values of the actual operation conditions x and y, respectively. n and m are the sequence numbers of each interval of the operation conditions x and y, respectively, where N and M are the total numbers of the interval. Δx and Δy represent the segmentation accuracy (segmentation criteria) of the operation conditions. The smaller the Δx and Δy, the more accurate the segmentation is and the closer it is to the actual situation. Still, the number of simulations will increase and lead to a decrease in simulation efficiency. Hence, it is necessary to choose the appropriate Δx and Δy to ensure that it is not out of line with the actual situation and to reduce the number of simulations effectively. $X_{s}$ is the value of the actual operation conditions corresponding to the train station. In this paper, there are two operation conditions, velocity and load, so x is used as the velocity operation condition and y as the load operation condition.

### 3.2. Simulation and Analysis Process

#### 3.2.1. Simulation of Temperature Field and Thermal Stress

**Figure 10:**
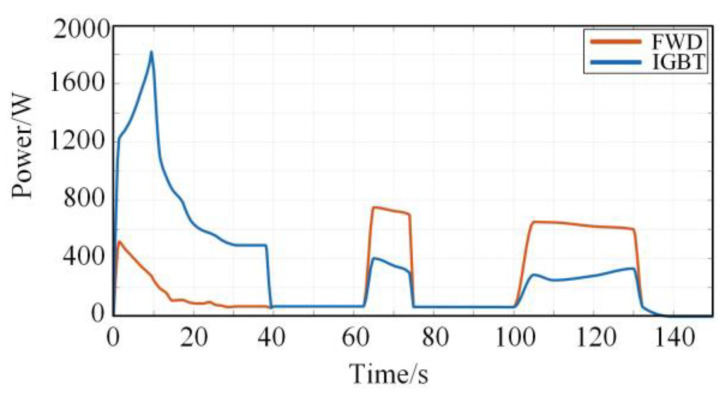
Power loss curve of IGBT and FWD.

This paper carries out the simulation based on the simplified operation conditions of StationB to StationC in Figure 7. The torque values corresponding to different rotational speeds are calculated, and input into the power loss data space is shown in Figure 5 and Figure 6. By fitting the power loss data output from the data space, the power loss curve of the IGBT and the FWD in the actual operation process can be finally obtained, as shown in Figure 10.

It is observed from Figure 10 that the power loss of the IGBT is higher than that of the FWD in the train traction phase. Furthermore, the power loss of the IGBT in the braking phase is lower than that of the FWD because the current is mainly distributed in the FWD during the braking phase. Therefore, the thermal stress generated by the power loss of the FWD may also accelerate the fatigue of the IGBT module’s internal material.

In order to improve the fidelity of the thermal stress simulation model, it is necessary to consider the fatigue damage of the IGBT module caused by the FWD power loss. Therefore, it is considered that the heat in the IGBT module mainly comes from the IGBT chip and the FWD chip. In this paper, we use the method of loading the heat generated internally onto the chip to analyze the thermal stresses generated in the module.

By integrating and averaging the power losses of IGBT and FWD in the whole simulation period in Figure 10, it is calculated that the average heating power of the IGBT and the FWD in this period is 450 W and 220 W, respectively. According to the dimensions of the IGBT and the FWD in Table 2, it is calculated that the heat generated inside the IGBT chip is 5.33 W/mm^3^ and the heat generated inside the FWD chip is 4.53 W/mm^3^.

During the simulation of the temperature field, the heat transfer mode between the whole module and the air is natural convection. In this paper, the natural convection heat transfer coefficient is 5 W·°C/m^2^. Because the thickness of each layer is minimal, the effect on the model’s overall heat dissipation can be ignored. Therefore, this paper only applies the convection heat transfer coefficient to the upper surface of the model. The heat-sink is ventilated between the fins, and the air volume is 2200 m^3^/h. In order to simulate the forced air cooling of the heat-sink, the convection heat transfer coefficient at the air duct of the heat-sink is set to 90 W·°C/m^2^ in consideration of the cross-sectional area of the air duct, the air-specific heat capacity, and the inlet and outlet temperatures.

**Figure 11:**
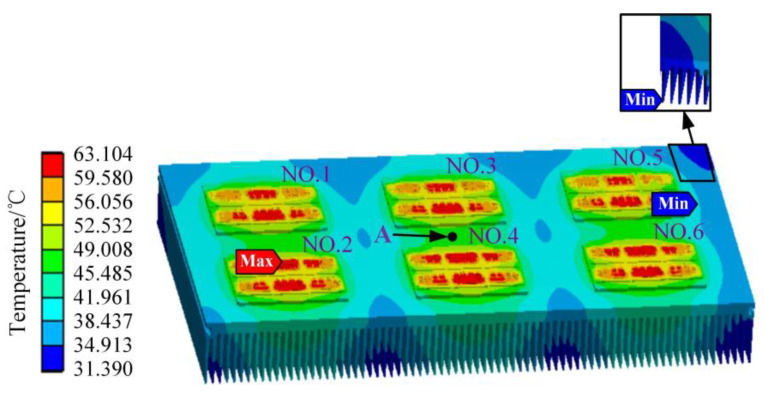
Temperature field of the IGBT module and the heat-sink in the traction converter.

The simulation results of the temperature field of the IGBT module and heat-sink in the traction converter are shown in Figure 11. The highest temperature of the whole model is 63.104 °C, which is located in the middle chip of the No. 2 IGBT module, and the heat is mainly concentrated on the chip and the bonding wire, and the lowest temperature is 31.39 °C at the fin at the edge of the heat-sink. For each IGBT module, the temperature distribution trend gradually decreases from the center to the surrounding, and the corner of the IGBT module has the lowest temperature. For the chips of the IGBT module, the high-temperature area of the chips in the middle position is large, while the high-temperature area of the chips on both sides is small, and the closer to the edge, the lower the temperature. Most of the heat is vertically transferred from each chip to the IGBT module substrate and presents a downward decreasing transfer trend.

Before coupling the temperature field results to the thermal stress field, the time is divided into 15 steps to apply the load. The lower surface of the IGBT module housing is fixed on the heat-sink, so a fixed constraint is added to the lower surface of the IGBT module. The fatigue failure of the IGBT module mainly occurs in the liner unit and above. This section focuses on the simulation of the liner unit and above in order to clarify the thermal stress in the actual operation.

**Figure 12:**
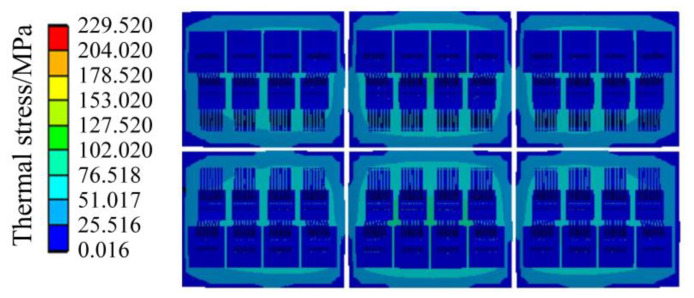
Thermal stress of the liner unit and above position.

**Figure 13:**
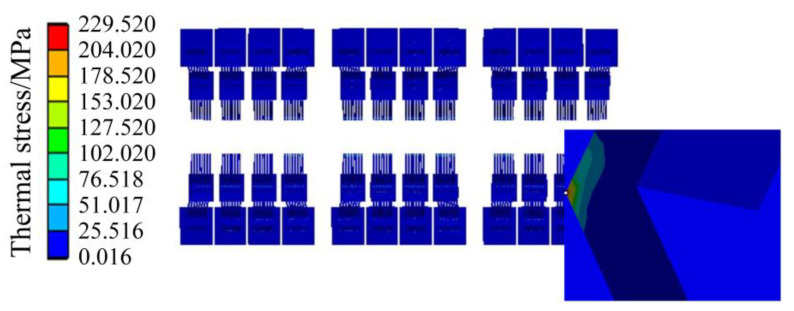
Thermal stress of the chip unit.

The thermal stress results of the IGBT module are shown in Figure 12 and Figure 13. Figure 12 shows the thermal stress of the liner unit and above position, and Figure 13 shows the thermal stress of the chip unit. As shown in Figure 12, the stress of the copper layer gradually decreases from the center to the periphery, which is the same as the distribution trend of the temperature field, indicating that the temperature distribution affects the stress distribution to a certain extent.

In Figure 11, the temperature of the chip and the bonding wire is very high. However, it is found that the stress of the chip and the bonding wire is small, as a whole, by observing Figure 12 and Figure 13, and the position where the thermal stress is large is distributed at the connection of the bonding wire and the chip. Because the thermal expansion coefficients of the bonding wire and the chip are different, the thermal stress at the connection between the bonding wire and the chip is significant, which conforms to the aging failure characteristics of the bonding wire.

**Figure 14:**
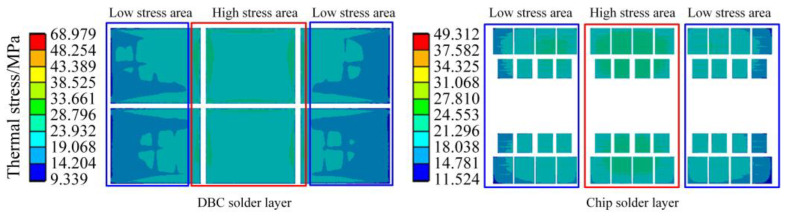
Thermal stress of the solder layer.

It can be seen that the maximum thermal stress of the DBC solder layer is 68.979 MPa, and the maximum stress of the chip solder layer is 49.312 MPa in Figure 14. As the location where the maximum stress value is located is most likely to cause fatigue failure compared to other locations, the authors have divided the high stress area and low stress area in Figure 14. The location of the maximum stress is in the high stress area, where the stress is relatively large and fatigue failure is more likely to occur. It can be seen from Figure 14 that the high stress areas of DBC solder layer and chip solder layer are concentrated in the middle of the whole module.

The modulus of elasticity can measure the ease of elastic deformation of different materials. Considering that the elastic modulus of DBC solder layer is the same as that of chip solder layer, theoretically, the maximum deformation of DBC solder layer under the maximum stress is 1.419 times that of chip solder layer, which indicates DBC solder layer is more prone to fatigue than chip solder layer.

When the temperature fluctuation of the IGBT chip ΔT_j_ is less than about 80 °C, thermal resistance at the joint of the chip gradually increases, and the bonding wire breaks afterward, which indicates the solder fatigue failure; when the ΔT_j_ is greater than about 100 °C, the bonding wire breaks without any increase in thermal resistance at the chip joints, which indicates a failure of the bonding wires lifting off [29]. In Figure 11, the maximum temperature of the IGBT module of the traction converter unit is 63.104 °C, which is less than 80 °C. Meanwhile, the modulus of elasticity of the solder layer and the bonding wire are 13.8 GPa and 83 GPa, respectively, from Table 4, and the maximum thermal stress between the bonding wires and the chip is 229.52 MPa from Figure 13. Therefore, the deformation of the DBC solder layer is 1.808 times of the bonding wire, and the deformation of chip solder layer is 1.292 times of the bonding wire. Combined with the mapping of thermal stress and the test results in Ref. [29], it can be concluded that the solder layer will fail before the bonding wire under the operation condition of the urban rail vehicle in Figure 7.

#### 3.2.2. Modal Analysis of IGBT Module

Modal analysis could search the resonance frequency and the corresponding vibration mode of the structure and arrange the frequencies from small to large. The real vibration mode of the structure is the superposition of each order of vibration mode. Generally, only low-frequency vibration is considered in the vibration of urban rail vehicles. In this paper, the natural frequencies of the free mode and the constrained state are simulated and calculated.

The parameters of each layer material are set according to the parameters of elastic modulus, Poisson’s ratio, and density in Table 4. Firstly, the natural frequency of the free mode is simulated and calculated. The calculation results are shown in Table 5. The first six modes are rigid body modes, and the others are elastic modes. The natural frequency of the rigid body mode is theoretically zero. Because there are some errors in the simulation calculation, the result is approximately zero.

In Table 5, the vibration modes of the first six orders are respectively translational along the x, y, and z axes and rotational around the x, y, and z axes. The vibration modes of the seventh to eleventh orders are respectively “∞” vibration along the Y axis, vertical bending along the Y axis, vertical bending along the X axis, “∞” vibration along the X axis, and wavy vibration.

**Figure 15:**
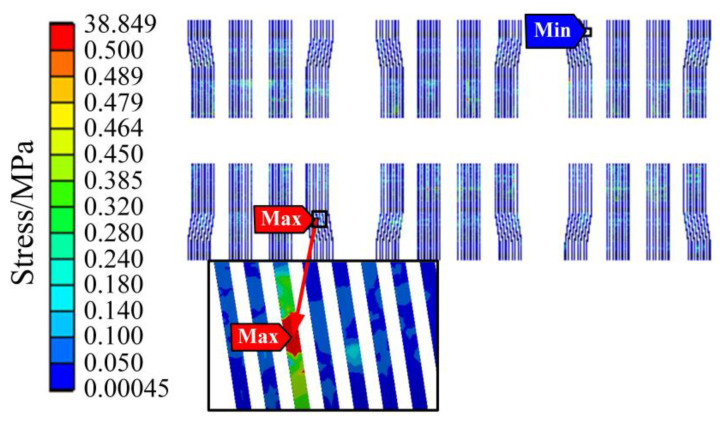
The stress of bonding wire under the first order mode.

**Figure 16:**
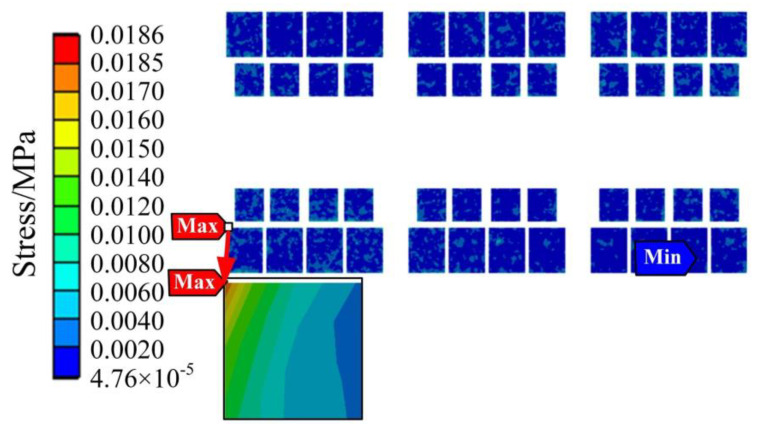
The stress of chip solder layer under the first order mode.

**Figure 17:**
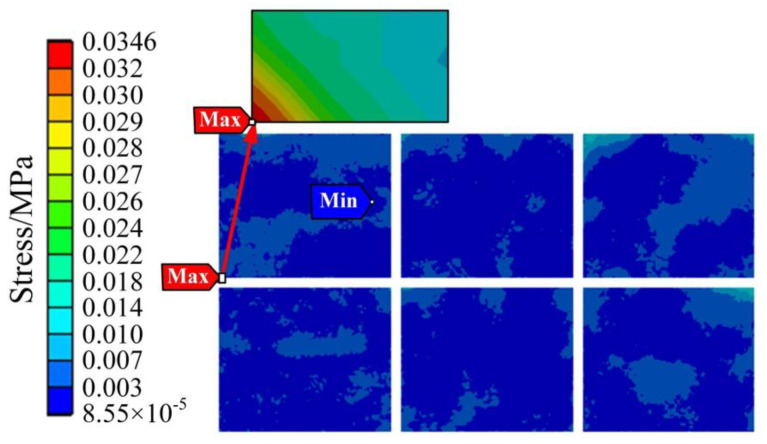
The stress of DBC solder layer under the first order mode.

The stress simulation results of bonding wire, chip solder layer, and DBC solder layer under the first order mode are shown in Figure 15, Figure 16 and Figure 17, respectively. It can be found that the vibration stress of the bonding wire is always greater than that of the solder layer.

Unlike the thermal stress, the maximum stress of the bonding wire in the first mode is 38.849 MPa, which is at the position where the bending degree of the bonding wire is relatively large. There is no other contact surface at this position, and the stability is poor, so the vibration dramatically impacts it. The maximum stress values of the DBC solder layer and chip solder layer are 0.0346 MPa and 0.0186 MPa, respectively, located at the corners of the solder layer. The phenomenon conforms to the fatigue characteristics of the solder layer. In addition, it is found that the vibration stress at most positions is minimal, and the vibration stress at only the local position is relatively large, whether in the bonding wire or the solder layer.

Under the condition of applying a fixed constraint on the bottom surface of the substrate, the natural frequency under the constraint condition is simulated and calculated. The calculation results are shown in Table 6. Because there are many bonding wires of the same structure, the modal difference in each order is slight, so the natural frequencies of each order are almost the same.

According to the analysis of free and constrained modal results, the minimum natural frequencies in the two states are 1985.8 Hz and 8639 Hz, respectively, and the natural frequencies are relatively high. Therefore, vertical resonance is not caused during vehicle operation [30].

#### 3.2.3. Vibration Stress Simulation

**Figure 18:**
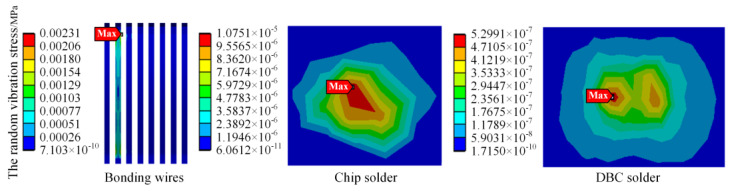
The random vibration stress of bonding wires, chip solder layer, and DBC solder layer.

After loading the power spectral density shown in Table 1 to the vertical direction of the IGBT module, the simulation was conducted on the vibration stress of the IGBT module under random vibration of the urban rail vehicle. The random vibration stress of module bonding wires, chip solder layer, and DBC solder layer are shown in Figure 18.

It is seen from Figure 18 that the 3σ equivalent maximum stress is located on the local bonding wire, with a value of 0.00231 MPa, which is far less than the yield strength (35 MPa) of silver. Therefore, elastic strain occurs on the bonding wire under random vibration load. In the meantime, the vibration stress of the bonding wires is the largest, and the vibration stress of the DBC solder layer is the smallest. This indicates that the bonding wires are more susceptible to random vibration stresses and more prone to fatigue damage under random vibration stresses than solder layer.

Since the bonding wire is subject to the largest vibration stress, to further clarify the deformation mechanism of the IGBT module bonding wire due to vibration, the local deformation of the bonding wire in the *x*-axis, *y*-axis, and *z*-axis directions is simulated.

**Figure 19:**
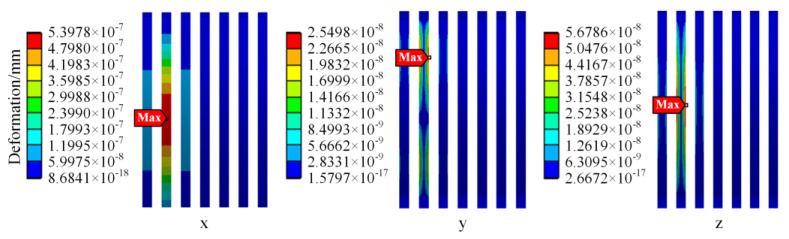
The deformation of bonding wires in different directions.

The simulation results of the deformation of the bonding wire in different directions are shown in Figure 19. Considering the installation of the IGBT module, the *x*-axis, *y*-axis, and *z*-axis of the IGBT module are defined in this paper to represent the train running direction, vertical direction, and left–right direction, respectively. The maximum value of the 3σ deformation in each direction is 5.3978 × 10^−7^ mm, 2.5498 × 10^−8^ mm, and 5.6786 × 10^−8^ mm, respectively. It can be found that although the train vibration is primarily vertical, the deformation of the bonding wire in the x direction (vehicle traveling direction) is the largest. In this paper, it is considered that the deformation of the bonding wire during the train operation is not only related to the vibration direction of the vehicle but also affected by the structure of the bonding wire.

## 4. Method Validation

### 4.1. Method Accuracy and Efficiency Validation

**Figure 20:**
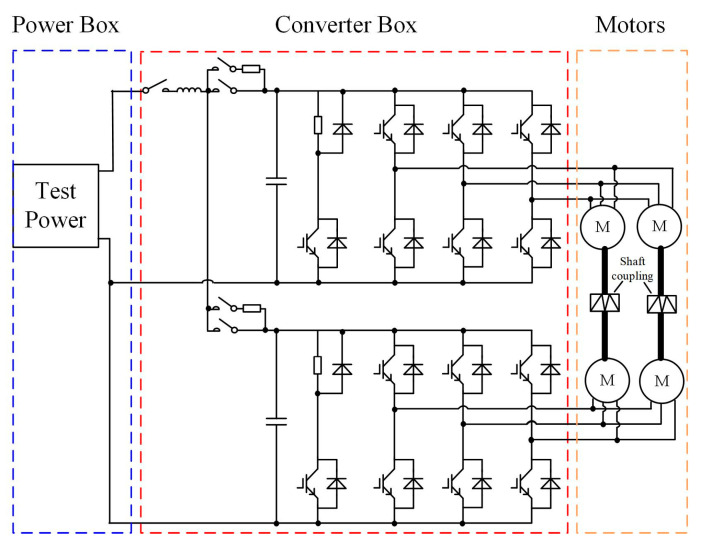
The back-to-back towing test platform topology.

**Figure 21:**
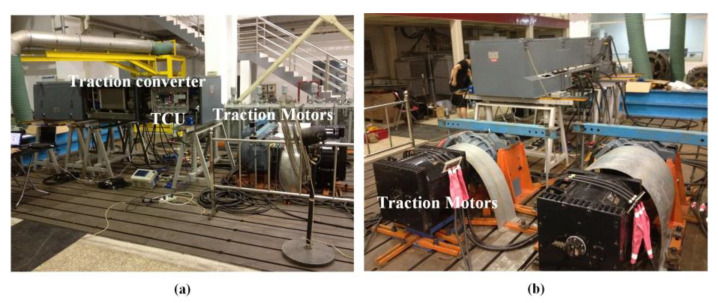
The back-to-back towing test platform with (**a**) overall view of the test platform and (**b**) side view of the test platform.

In order to verify the validity of the established finite element model (FEM) thermal simulation results, an experiment platform was established, as shown in Figure 20 and Figure 21. The platform mainly consists of a traction converter (DC-link capacitor, inverter circuit, and braking circuit, et al.), traction control unit (TCU), traction motors, and a cooling system. The platform parameters are illustrated in Table 7.

**Figure 22:**
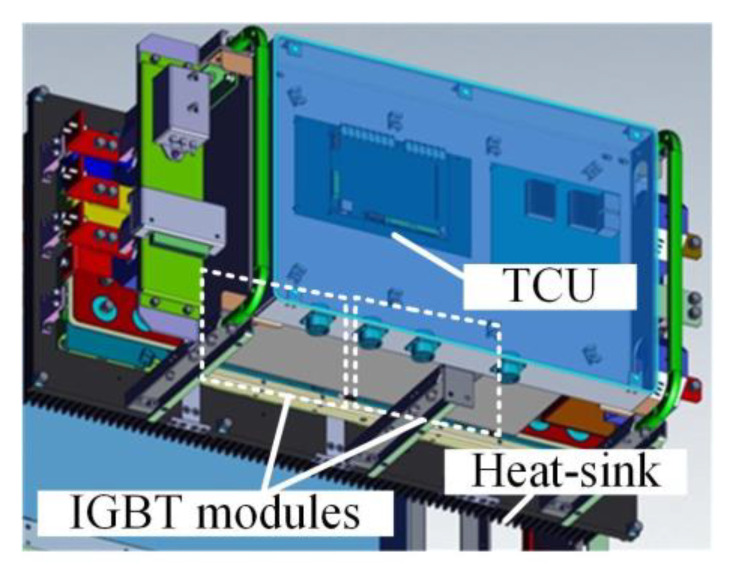
Traction converter 3D structure.

**Figure 23:**
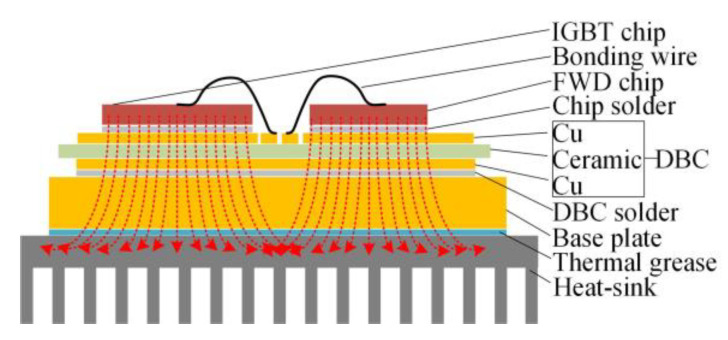
The heat conduction path of the chip in the module.

The traction converter in Figure 21 is used to control the traction motors according to the speed and load profile in the metro plan map of a line in S city, China. Considering that six IGBT modules in the traction converter mentioned in our previous study [31] are compact, fixed on the heat-sink and the TCU, sensor, and other devices are installed in front of the IGBT modules, as shown in Figure 22, it is difficult to measure the chip junction temperature inside the IGBT module. In practical applications, the IGBT module is mechanically fixed on the heat-sink to increase the heat dissipation efficiency [32]. The heat conduction path of the chip in the module is shown in Figure 23. The heat is eventually transmitted to the heat-sink, so the temperature of the heat-sink depends on the temperature of the chip, which can indirectly reflect the change in the chip temperature to a certain extent. Therefore, considering the actual test conditions of temperature measurement and better reflecting the thermal effect brought by the IGBT module, the temperature measurement point is chosen to be located in the middle of the two IGBT modules on the heat-sink, corresponding to pointA in Figure 11.

**Figure 24:**
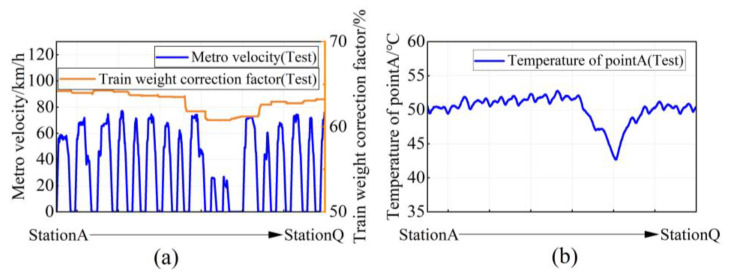
The results of the experiment with (**a**) the curve of the vehicle operation conditions and (**b**) the temperature of pointA in the test.

In order to simulate the actual train situation, the test was conducted according to the operation planning and load situation, and the test waveform diagram was selected to simulate StationA to StationQ. The typical train speed situation and the percentage change in train equivalent load during the test are shown in Figure 24a.

It can be seen that during the trip of StationA to StationQ, the train runs with different loads and different speeds between stations. In this paper, Δx is set to 10 and the minimum value of the actual speed operation condition is the corresponding speed from StationJ to StationK, which is 26 km/h. N is taken as 5 according to Equation (6). In the process from Station A to Station Q, the speed limit is 80 km/h and the uniform speed operation conditions *X_s_*_1_, *X_s_*_8_, *X_s_*_13_ from StationA to StationB, StationH to StationI, and StationM to StationN are 57.2 km/h, 62.4 km/h, and 60.55 km/h, all within the range of [56,66), marked as v1. Similarly, the corresponding speeds *X_s_*_2_~*X_s_*_7_, *X_s_*_9_, *X_s_*_12_, *X_s_*_14_~*X_s_*_16_ from StationB to StationH, StationI to StationJ, StationL to StationM, and StationO to StationQ are all within the range of [66,76), marked as v2. The speeds *X_s_*_10_, *X_s_*_11_ from StationJ to StationL are in the range of [26,36), marked as v3.

For the load condition segmentation, this paper sets Δy to 0.9%, and the minimum value of the actual load condition is 60.8%. M is taken as 4 according to Equation (6). Therefore *Y_s_*_1_~*Y_s_*_8_ in the range of [63.5%, 64.4%) is marked as Load1; *Y_s_*_9_ in the range of [61.7%, 62.6%) is marked as Load2; *Y_s_*_10_~*Y_s_*_12_ in the range of [60.8%, 61.7%) is marked as Load3; and *Y_s_*_13_~*Y_s_*_16_ in the range of [62.6%, 63.5%) is marked as Load4.

The whole line is segmented according to the same speed and load condition interval combinations [*X_sk_*, *Y_sk_*] at each station. The whole line in this paper can be segmented into seven intervals. Interval1 includes StationA–StationB and StationH–StationI; interval2 includes StationB–StationH; interval3 includes StationI–StationJ; interval4 includes StationJ–StationL; interval5 includes StatonL–StationM; interval6 includes StationM–StationN; and interval7 includes StationN–StationQ.

The average values of the speed and load conditions for each OIS of the whole line are calculated to obtain simplified operation conditions among the stations, the results of which are shown in Table 8. Finally, the operation process of 17 stations is segmented into 7 different operation condition intervals.

The corresponding temperature change in heat-sink pointA during operation is shown in Figure 24b. It can be seen that the temperature of the heat-sink pointA fluctuates at about 51 °C during high-speed operation with passengers. However, the temperature drops very quickly during the terminal and departure stations (StationJ–StaitonL) because the speed and load are the lowest.

**Figure 25:**
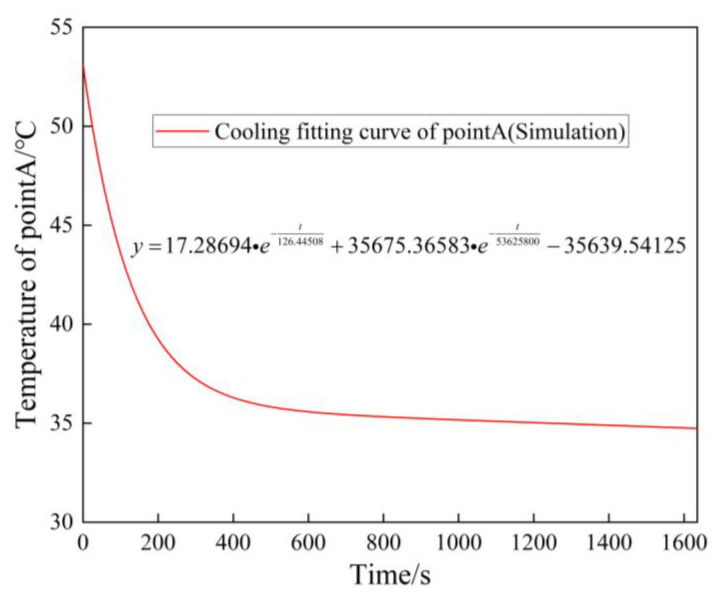
Cooling fitting curve of pointA.

**Figure 26:**
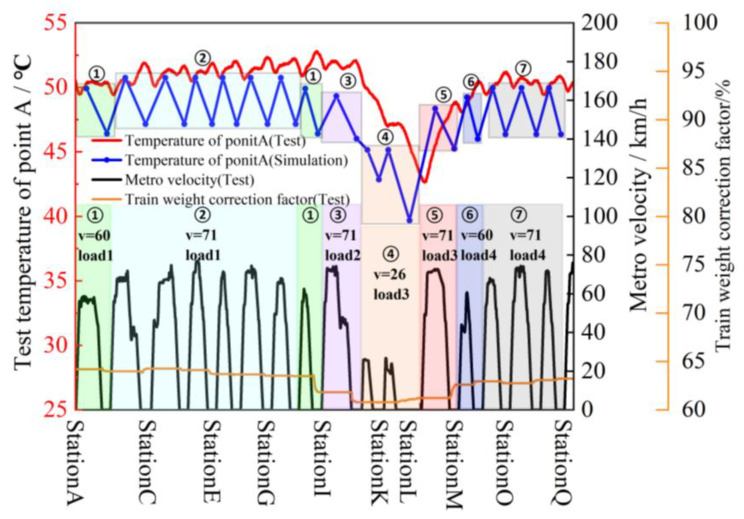
Comparison of simulation and experimental results.

Based on the power loss data space obtained in Section 2, the average power loss in the seven operating condition intervals are mapped. Then, the temperature and thermal stress fields of the traction converter are obtained according to the finite element simulation method introduced in Section 2, and the stable temperature at pointA of the heat-sink is extracted from them. At the same time, the temperature change at pointA of the heat-sink corresponding to the initial stable temperature and stopping time of the seven operating interval is obtained by the cooling curve shown in Figure 25. Thus, the temperature simulation curve, experimental measurement results and operating conditions at pointA of the heat-sink are shown in Figure 26. It can be seen that the proposed simplified interval segmentation simulation method achieves good results. The temperature change trend of the simulation result of the heat-sink pointA temperature can follow the actual test temperature well and can reflect the actual temperature change better. Since the simulation results of the operating stable temperature are recorded at the midpoint of time between adjacent stations, and the simulation results of cooling stable temperature are recorded at the midpoint of stopping time of each station, the simulation temperature curve lags behind the actual temperature curve. The simulation results of cooling in seven intervals are significantly lower than the values after the actual temperature reduction, because the overall temperature of the converter is higher than the air temperature during the actual operation, and the surrounding environment temperature also rises, resulting in slower heat consumption, which makes the actual cooling effect weaker, but the error between the simulation results and the actual tested cooling change value is 1.5~2 °C, which has little impact on the temperature evaluation and subsequent lifetime calculation.

In addition, the efficiency of the proposed method needs to be discussed. Simulation of the conditions described in this paper using the traditional method requires simulation of each station for a total of 16 temperature simulations of the operating process and calculation of the temperature results of 16 cooling processes. Using the proposed method, only seven temperature simulations of the operation process and eight temperature results of the cooling process are required.

Through the comparison and discussion of the actual test and simulation results, it can be shown that the proposed simplified method of segmenting line intervals in simulation can effectively reduce the simulation time, improve the research efficiency, and help analyze the temperature and thermal stress of the IGBT module of the whole line under the condition that the simulation results of the heat-sink pointA temperature are not significantly different from the actual test results.

### 4.2. Method Application

After verifying the simulation results and the effectiveness of the proposed method, the proposed method is used to give further cases of lifetime calculation based on the train line studied in this paper. The proposed method is used to build an electric power simulation model in Matlab, analyze the condition of each station to segment the interval, obtain the average power loss of each interval, then simulate on Ansys to obtain the IGBT stress of each interval, and, finally, calculate the cumulative damage degree of the IGBT of the whole line to obtain the lifetime of the IGBT module.

IGBT modules in traction converters are mainly subjected to thermal and random vibration stresses, which cause partial fatigue damage to the IGBT modules when cycled during prolonged operation. Comparing Figure 12, Figure 14 and Figure 18, it is found that the vibration stress is much lower than the thermal stress, and the quantity difference between the two kinds of stresses is, so far, that the vibration stress has very little impact on the IGBT lifetime. It can be considered that the main stress source of module failure is thermal stress, and the damage brought by the main thermal stress to the IGBT module is considered in the process of lifetime calculation. During the operation of the IGBT, both the solder layer and the bonding wire are damaged by thermal stress, so the lifetime of the IGBT module can be calculated by the linear fatigue damage cumulative theory of Miner’s law, which is expressed in Equation (7).

(7) $$D=\sum_{i=1}^{k}\frac{n_{thi}}{N_{fthi}}$$

In Equation (7), *k* is the number of intervals to be segmented, D is the total degree of damage produced by various stresses per unit time, $n_{thi}$ is the number of stations in the *i*th interval, and $N_{fthi}$ is the maximum number of cycles corresponding to the thermal stress of the *i*th interval operation condition.

Considering that the aging of the solder layer starts with the reduction of area at the relative maximum stress near the generated void and gradually expands to create a void, the relative maximum stress will keep acting at the tip of the void and the relative maximum stress value will increase as the aging degree deepens [33]. In the actual application of the IGBT, the solder layer generates cavities in different places during the process of solder layer fatigue. In this paper, the maximum number of cycles is calculated using the maximum thermal stress of the solder layer in each interval.

Based on the lifetime assessment model, considering elastic strain proposed by Manson and Coffin [34], and linearly correcting it for elastic stress, the calculation model for the maximum number of cycles corresponding to different stresses is obtained, shown in Equation (8).

(8) $$\{\begin{array}{l} \frac{\Delta \varepsilon_{e}}{2}=\frac{\left(\sigma_{f}^{\prime }-\sigma_{m}\right)}{E}{\left(2N_{f}\right)}^{b}\\ \sigma_{m}=E\Delta \varepsilon_{e} \end{array}$$

In Equation (8), $N_{f}$ is the maximum number of cycles corresponding to different stresses, $\Delta \varepsilon_{e}$ represents the elastic strain, $\sigma_{f}^{\prime }$ is the fatigue strength coefficient, which is generally expressed as 3.5 times the tensile strength [35], $\sigma_{m}$ is the fatigue stress, E is the modulus of elasticity, and b is the fatigue strength index. Ref. [35] summarizes the fatigue test results of a variety of metallic materials, concluding that the fatigue strength index b=−0.12. The elastic modulus of the solder layer is 13.8 GPa and that of the bonding wire is 83 GPa. The tensile strength of the solder layer is 75, it can be calculated that the $\sigma_{f}^{\prime }$ of solder layer is 262.5.

Take the simulation in the Section 2 as an example to calculate the maximum cycle number $N_{fth2}$ and damage degree $D_{2}$ corresponding to the stress of line interval2 segmented by the proposed method. As shown in Figure 14, the maximum thermal stress is 68.9 MPa, which is recorded as the maximum thermal stress of interval2. Line interval2 includes six station intervals, and $n_{th2}$ is recorded as 6. The thermal stress of solder layer $\sigma_{m}{}_{th2}$ used to calculate the maximum number of cycles is 68.9 MPa. The maximum number of cycles of the solder layer under thermal stress $N_{fth2}$ is 884,310 by the calculation of Equation (8). Therefore, $D_{2}$ can be calculated as 6.78495 × 10^−6^ by Equation (7).

Similarly, the maximum number of cycles and damage degree can be calculated for other segmentation intervals. Thus, the IGBT module cumulative damage degree D of a line can be finally calculated by Equation (7) as 1.01635 × 10^−5^.

When the cumulative damage degree equals 1, the fatigue lifetime of the IGBT module is considered to be run out. The train could run 98,391.5 times according to the operation condition of the line; based on 10 round trips per day (20 one-way trips) and 330 days of operation per year, the IGBT module of the line could run for 14.91 years.

By statistically counting the number of damaged IGBT modules in vehicles of this line, the IGBT module of one traction converter of this vehicle has two failures during 2017–2022, and the failures in time (FIT) λ of the IGBT modules in one converter can be calculated by Equation (9) as is 1/(157,680 h).

(9) $$\lambda =\frac{r}{n\cdot t}=\frac{2}{6\times 6\times 365\times 24}=\frac{1}{157,680h}$$

In Equation (9), n is the total number of IGBT modules in regular operation, t is the total operation time, and r is the number of IGBT modules that failed during the operation time t for n regular modules.

FIT is equivalent to MTBF as Equation (10).

(10) $$\mathrm{MTBF}=\frac{1}{\lambda }=18\mathrm{years}$$

It can be found that the MTBF calculated by the actual statistics is 18 years, and the lifetime calculated by the method proposed in this paper is 14.91 years. The error in the value between the calculated and actual statistics of the lifetime is 3.09 years, and this paper considers that the sources of error can be summarized as follows: (1) Random fluctuation of one-way passenger volume (the main reason): a one-way line operation condition is used in the simulation and calculation process, while the passenger flow (load) of each one-way trip in the actual process is uncertain, so the difference between the actual randomly changing one-way load and the simulated one-way load is the main reason for the lifetime calculation error. (2) Operation and maintenance time interval (O and M): a reasonable O and M time interval setting will, to a certain extent, help workers to detect problems in time and help to improve the IGBT module lifetime. (3) Vehicle operation terrain and other environmental factors: when the road surface is a slope, the converter output is different during the up train travel and down train travel. For the above reasons, this paper intends to take into account the random distribution of one-way load variation, the O and M time interval, and the operating environment in the subsequent research to form the lifetime influence factor and use it in the process of calculating the lifetime. The influence factor is corrected and iterated by comparing the calculated lifetime with the actual statistical lifetime results, so that a more accurate lifetime calculation can be achieved. Therefore, the lifetime calculation method based on the simulation of line interval segmentation proposed in this paper has a certain reference value and can assist in guiding the development of the maintenance plan of traction converters in urban rail vehicles.

## 5. Conclusions

In this paper, a simplified condition evaluation simulation method and lifetime calculation method based on segmenting operating intervals is proposed for traction converter IGBT modules in urban rail vehicles. Based on the similarity analysis of the average power loss between each station of the line, the interval of the actual line is segmented, and the simulation analysis process of the temperature and stress fields of the IGBT module is introduced for one of the intervals. The magnitude and distribution of bonding wire and solder layer temperature, thermal, and vibration stress within the IGBT module are analyzed. The segmentation of the line operation interval is completed according to the proposed method and the line speed and load reproduction tests are carried out by the test platform. The effectiveness of the line interval segmentation simulation of the proposed simplified method is verified by the heat-sink temperature, which could guarantee the simulation accuracy and improve the efficiency by interval segmentation compared with the traditional method, showing that the temperature change at lower frequencies can also describe the temperature change trend of the device during the whole line operation. To further improve the applicability of the method in practical applications, the lifetime of the traction converter IGBT module is calculated and verified by the thermal stresses in the various segmented intervals. The method simplifies the line operation conditions and could be used to efficiently evaluate the condition of IGBT modules, heat-sinks, and other components of traction converters in urban rail vehicles during operation across the whole line, which is an auxiliary guide for the maintenance work of traction converters.

## Figures and Tables

**Table 1:** Random vibration power spectral density (PSD).

Frequency (Hz)	PSD Grade (g^2^/Hz)
5	0.00002
17	0.001
40	0.001
50	0.01
60	0.01
70	0.001
150	0.001
200	0.0005
500	0.0005

**Table 2:** IGBT module dimensions.

Internal Unit	Material Composition	Dimension (mm)
Bonding wire	Ag	0.4 (diameter) 1.3 (arc height)
IGBT chip	Si	15 × 12.5 × 0.45
FWD chip	Si	11 × 9.8 × 0.45
Solder layer 1	SnAg	15 × 12.5 × 0.1 (IGBT) 11 × 9.8 × 0.1 (FWD)
Copper layer 1	Cu	55 × 46 × 0.3
Liners	AlN	58 × 49 × 0.8
Copper layer 2	Cu	58 × 49 × 0.3
Solder layer 2	SnAg	58 × 49 × 0.4
Substrate	Al-SiC	187.5 × 137 × 4.7

**Table 3:** Heat-sink dimensions (mm).

Length	Width	Substrate Thickness	Tooth Length	Tooth Thickness	Tooth Pitch
920	450	16	72	3.36	12

**Table 4:** Material properties of each layer.

Layer	Density (kg·m^−3^)	Thermal Conductivity (W·(m·K)^−1^)	Coefficient of Thermal Expansion (10^−6^·K^−1^)	Specific Heat Capacity (J·(kg·K)^−1^)	Elastic Modulus (GPa)	Poisson’s Ratio
Bonding wire	11,000	429	19	232	83	0.37
Chip layer	2330	118	2.9	690	130	0.28
Solder layer	7360	33	30	180	13.8	0.35
Copper layer	5361	200	7.4	519	225	0.29
Substrate	2960	220	7.9	741	741	0.22
Liner	3220	150	3.5	740	325	0.31
Heat-sink	2689	273.5	23	951	71	0.33

**Table 5:** The natural frequency of free mode.

Orders	Frequency (Hz)
1	0
2	0
3	0
4	0.0015
5	0.0092963
6	0.011533
7	1985.8
8	2130.3
9	4454
10	4568.6
11	5609.8

**Table 6:** The natural frequency under the constraint condition.

Orders	Frequency (Hz)
1	8639
2	8639
3	8639.8
4	8639.9
5	8640
6	8640.2
7	8640.4
8	8640.4
9	8640.5
10	8640.8
11	8640.9

**Table 7:** Parameters of the experiment platform.

Parameter	Value
DC Voltage	1.5 kV
DC-link capacitor in a converter	4300 μF
IGBT	FZ1500R33HE3
Switch frequency	450 Hz
Traction motor nominal/maximum current	132 A/271 A
Traction motor nominal power	210 kW
Traction motor nominal/maximum speed	1800 rpm/3472 rpm
Traction motor maximum traction force	1630 Nm
Traction motor nominal displacement factor	0.85
Cooling system	Forced air cooling
Oscilloscope	DL750
Chopper diode	FZ1500R33HE3

**Table 8:** Line interval segmentation results.

Interval	Interval1	Interval2	Interval3	Interval4	Interval5	Interval6	Interval7
Velocity	60	71	71	26	71	60	71
Load	63.87%	63.87%	61.83%	60.9%	60.9%	62.85%	62.85%

## Data Availability

The data that support the findings of this study are available from the corresponding author upon reasonable request.
